# Comparison of ABR and ASSR using narrow-band-chirp-stimuli in children with cochlear malformation and/or cochlear nerve hypoplasia suffering from severe/profound hearing loss

**DOI:** 10.1007/s00405-021-06990-4

**Published:** 2021-07-27

**Authors:** Katharina Eder, Daniel Polterauer, Sebastian Semmelbauer, Maria Schuster, Tobias Rader, Eva Hoster, Wilhelm Flatz

**Affiliations:** 1 Department of Oto-Rhino-Laryngology, Head and Neck Surgery, Ludwig-Maximilians-University Munich, Marchioninistr. 15, 81377 Munich, Germany; 2 Institute of Medical Informatics, Biometry, and Epidemiology, Ludwig-Maximilians University Munich, Munich, Germany; 3 Department of Radiology, Ludwig-Maximilians-University Munich, Munich, Germany

**Keywords:** Cochlear malformation, Nervus cochlearis hypoplasia, Hearing threshold estimation, ASSR, ABR, NB chirp

## Abstract

**Objectives:**

In pediatric audiology, objective techniques for hearing threshold estimation in infants and children with profound or severe hearing loss play a key role. Auditory brainstem responses (ABR) and auditory steady-state responses (ASSR) are available for frequency-dependent hearing threshold estimations and both techniques show strong correlations but sometimes with considerable differences. The aim of the study was to compare hearing threshold estimations in children with and without cochlear and cochlear nerve malformations.

**Methods:**

Two groups with profound or severe hearing loss were retrospectively compared. In 20 ears (15 children) with malformation of the inner ear and/or cochlear nerve hypoplasia and a control group of 20 ears (11 children) without malformation, ABR were measured with the Interacoustics Eclipse EP25 ABR system^®^ (Denmark) with narrow-band CE-chirps^®^ at 500, 1000, 2000 and 4000 Hz and compared to ASSR at the same center frequencies under similar conditions.

**Results:**

ABR and ASSR correlated significantly in both groups (*r* = 0.413 in malformation group, *r* = 0.82 in control group). The malformation group showed a significantly lower percentage of “equal” hearing threshold estimations than the control group. In detail, patients with isolated cochlear malformation did not differ significantly from the control group, whereas patients with cochlear nerve hypoplasia showed significantly greater differences.

**Conclusion:**

ABR and ASSR should be used jointly in the diagnostic approach in children with suspected profound or severe hearing loss. A great difference in hearing threshold estimation between these techniques could hint at the involvement of cochlear nerve or cochlear nerve hypoplasia itself.

## Introduction

The therapeutic strategy in infants and children with severe-to-profound hearing loss is based on hearing threshold estimations by frequency-specific auditory brainstem responses (ABR) in combination with behavioral measures and other diagnostic tools. Also, auditory steady-state responses (ASSR) offer frequency-dependent hearing threshold estimation and came into play [1, 2]. Both methods are widely discussed, compared to each other, and referenced towards behavioral hearing threshold estimations in the literature. In general, ABR and ASSR show good correlations in hearing threshold estimations [3–14]. However, stimuli and methodological setups are partly varying, comparison and validation remains challenging for individual clinical application and will be topic of future research.

ASSR has been reported to show advantages for the threshold detection in profound hearing loss in cochlea implant candidates [15] and shows good consistency with behavioral hearing thresholds in school children and adolescents with an increased sensitivity especially for higher degrees of hearing loss [16]. This study with 10 children (20 ears) concluded that ASSR is a valuable tool to detect residual hearing, and in some cases, may be the only method to accurately characterize residual hearing for profound hearing impairment in children, where reliable behavioral responses cannot be obtained [16]. Nevertheless, ASSR could not establish as single “gold-standard” for hearing threshold estimation in infants and children with severe-to-profound hearing loss to date.

Until now, it has not been examined, whether temporal bone or hearing nerve malformations might have an influence on threshold estimations by ABR or ASSR. Approximately 20% of patients with congenital hearing loss show inner ear malformations that can be diagnosed by radiology techniques, and the majority of these patients suffer from profound or severe hearing loss [17]. Inner ear malformations are classified according to radiologic findings into Michel deformity, cochlear aplasia or hypoplasia, common cavity, incomplete partition (IP) I, II, and III, and enlarged vestibular aqueduct with varying other findings regarding the vestibule, semicircular canals, internal auditory canal, vestibular aqueduct and cochlear nerve [17, 18].

In this study, we compared hearing threshold estimation derived with narrow-band CE chirps evoked ABR and ASSR in children with radiologically proven cochlear malformation (CM), cochlear nerve hypoplasia (CNH) or combined malformation with children without temporal bone and inner ear pathology, both groups suffering from profound or severe hearing loss.

## Materials and methods

### Patient data

We retrospectively evaluated data from 20 ears of 15 children (7 females, 8 males) with an age from 9 months to 7 years with cochlear malformation or/and hearing nerve hypoplasia proven by magnetic resonance imaging (MRI) and/or computer tomography (CT) (see below) and profound or severe hearing loss that was obtained between 07/2014 and 01/2018. As control group, we evaluated 20 ears of 11 children (6 females, 5 males) with an age from 8 months to 6 years without pathological findings in MRI/CT and profound or severe hearing loss. In all children hearing threshold estimations had been obtained by ABR and ASSR measurements because newborns did not pass hearing screening, acoustic hearing testing was pathologic not only due to middle ear effusions, or for follow-up in children with known hearing loss. Only patients with hearing threshold ≥ 70 dB in at least two frequencies in ABR measurements were included (hearing threshold values in remaining frequencies were better in individual cases). Premature infants and infants with known neurological disorders were excluded. One patient with cochlear nerve hypoplasia showed detectable otoacoustic emissions.

The use of data is in accordance with ethical principles stated in the Declaration of Helsinki and was approved by the local ethics committee and the local data protection commissioner (Project No. 17-448).

### ABR and ASSR procedure

To ensure comparable conditions in ABR and ASSR measurements for retrospective evaluation of both methods, only data sets that were performed at the same day during general intravenous anesthesia were included in the study. Anesthesia had been necessary that day to obtain MRI and CT scans. Measurements were acquired consecutively in a noise-absorbing room which was electrically shielded in.

For both methods standard narrow-band CE-chirps^®^ with center frequencies (CF) at 500 Hz, 1000 Hz, 2000 Hz, and 4000 Hz were used [4]. Measurements were performed consecutively with the Interacoustics Eclipse EP25 ABR system (Middelfart, Denmark). It used calibrated outputs with Etymotic Research Eartone 3A ABR insert earphones (Elk Grove Village, USA). Surface recording electrodes were positioned on the high forehead, both mastoids, and low forehead. The impedance between the electrodes was kept below 2 kΩ. The hardware high-pass filter of the eclipse was set to 100 Hz with a slope of 12 dB per octave. This cutoff frequency prevents interferences from the mains power supply. To reduce high-frequency interferences, which are outside the range of interest, the signal was low-pass filtered with a cutoff frequency of 3000 Hz. In ABR measurements, the stimulus was repeated at a rate of 44.1 Hz. The recordings were taken in a time frame between 0 and 15 ms and evaluated in a time frame between 4.5 and 14 ms. Recordings were rejected if the absolute value of the electroencephalography (EEG) signal amplitude exceeds 40 µV. The acceptance criteria of a specific frequency and level combination were either 4000 collected EEG recordings or a residual noise level beneath 30 nV. To reduce artifacts from the contralateral ear, the contralateral masking level was set 30 dB below stimulation level. In the ABR technique, hearing threshold estimation was based on the detection of reproducible Jewett wave V at above-mentioned CFs [23]. All ABR measurements were analyzed by two experienced audiologists. In detail, in ABR measurements, the individual waveform was first determined at 80 dB nHL. In case of reproducible wave V, we successively reduced the stimulus amplitude by 10 dB decrements until wave V disappeared. After roughly estimating the threshold in this way, steps were reduced to 5 dB for more precise resolution. If there was no reproducible wave V at 80 dB nHL, ABR measurements were continued at 90 and 100 dB nHL, respectively. After obtaining ABR thresholds, ASSR were measured. For ASSR measurements, the multiple auditory steady-state response (MASTER) technique of the Interacoustics Eclipse Software was used. The stimuli were modulated and presented at a repetition rate of around 90 Hz (setting of the system). The recordings were also rejected if the absolute value of the residual noise level of the EEG was above 40 µV. Automatic threshold correction of the software was not used, because two independent techniques should be compared irrespective of individual correction factors. ASSR responses were collected at both ears, and all CFs simultaneously starting at 80 dB nHL according to ABR measurements. The response was marked as positive if it reached an amplitude level within 95% confidence interval during the default testing time of 6 min. If the positive response was detected by the algorithm earlier than the 6 min, the data acquisition was stopped, and the stimulus intensity was decreased by 10 dB. If the confidence interval of response was less than 50% within 3 min, the measurement was stopped, and the test was repeated with a 5 dB increased stimulus intensity. In children with single-sided hearing impairment, ASSR measurements were only performed in the impaired ear with a standard masking level of 70 dB on the contralateral side.

The imputing technique was used for statistical reasons: in data sets without a measurable response up to a stimulus level of 100 dB nHL, the hearing threshold was set to 110 dB nHL and therefore, 110 dB nHL was used for calculations.

### Magnetic resonance imaging (MRI) and computer tomography (CT)

Magnetic resonance imaging (MRI) was performed on a 3T scanner (Siemens MAGNETOM Skyra, Erlangen, Germany). The MRI examination protocol included diffusion-weighted-imaging (DWI), T2w-imaging, T1w-imaging pre- and post-gadolinium application, as well as high-resolution 3D T2-SPACE imaging with high-frequency-pulse transmit technique (ZOOMit), which is a single slab 3D TSE sequence with slab selective, variable excitation pulse (flip angle evolution-sequence). The latter MRI technique relies on fast turbo spin imaging sequences with isotropic resolution allowing for equal reconstruction in all planes. In our study voxel size was 0.5 × 0.5 × 0.5 mm, echo train length 54, repetition time 100 ms, echo time 125 ms, flip angle 100, bandwidth 255, 100% sampling, acquisition matrix 320 × 164, number of averages 2. In cases where 3T imaging was not available, patients were scanned on a 1.5T scanner (Siemens Magnetom Aera, Erlangen, Germany) using an isotropic high-resolution three-dimensional strongly T2-weighted sequence (3D constructive interference in steady-state, 3D-CISS) with a voxel size of 0.5 × 0.5 × 0.5 mm, echo train length 1, repetition time 6.3 ms, echo time 2.81 ms, flip angle 62, bandwidth 420, 100% sampling, acquisition matrix 384 × 288, number of averages 2.

Computer tomography (CT) was performed on a Dual-Source CT-scanner (Siemens SOMATOM Definition Flash), a 2 × 128 row CT scanner with a rotation time of 0.28 s, using a dedicated temporal bone scan protocol with a slice thickness of 0.75 mm, 120 kV, variable mAs (111–151 mAs), including selective reconstruction of each temporal bone using a UHR-kernel (V80u3) in three planes.

Inner ear malformations were classified as outlined in the introduction according to Sennaroglu et al. [17, 18] by two independent radiologists. Patients/ears were only grouped by CM, CNH and combined CM and CNH, due to the limited number of patients/ears.

### Data analysis

Data analysis was performed with Excel (Microsoft, Redmond, WA, USA) and SigmaPlot (Jandel Corp., San Rafael, CA, USA). Correlations between hearing thresholds of the two methods were calculated by Spearman Rank Order Correlation since data failed normality testing. A *p* value < 0.05 was considered significant. Differences between malformation patient collectives and control group on an individual value basis were compared by Chi-Square Test. Subgroups of the malformation patient collective itself and control group were compared as “mean of Δ ABR–ASSR (in dB)” and “no. of outliers”, defined as no. of CFs, where Δ ABR–ASSR ≥ 15 dB, by Mann–Whitney-Rank Sum-Test and Kruskal–Wallis-One Way Analysis. In addition, all pairwise multiple comparison procedure (Dunn’s Method) was applied.

## Results

Measurements were performed on 20 ears (8 females, 7 males) with radiologically proven CM and/or CNH and on a control group in 20 ears without pathology of the temporal bone. In some ears ABR measurements were not performed for the CF of 500 Hz due to limited time and anesthesia reasons. The malformation group had a mean age of 34 months (range 7 months–7 years) at the day of hearing threshold estimation, the control group a mean age of 33 month (range 8 months–6 years).

**Fig. 1:**
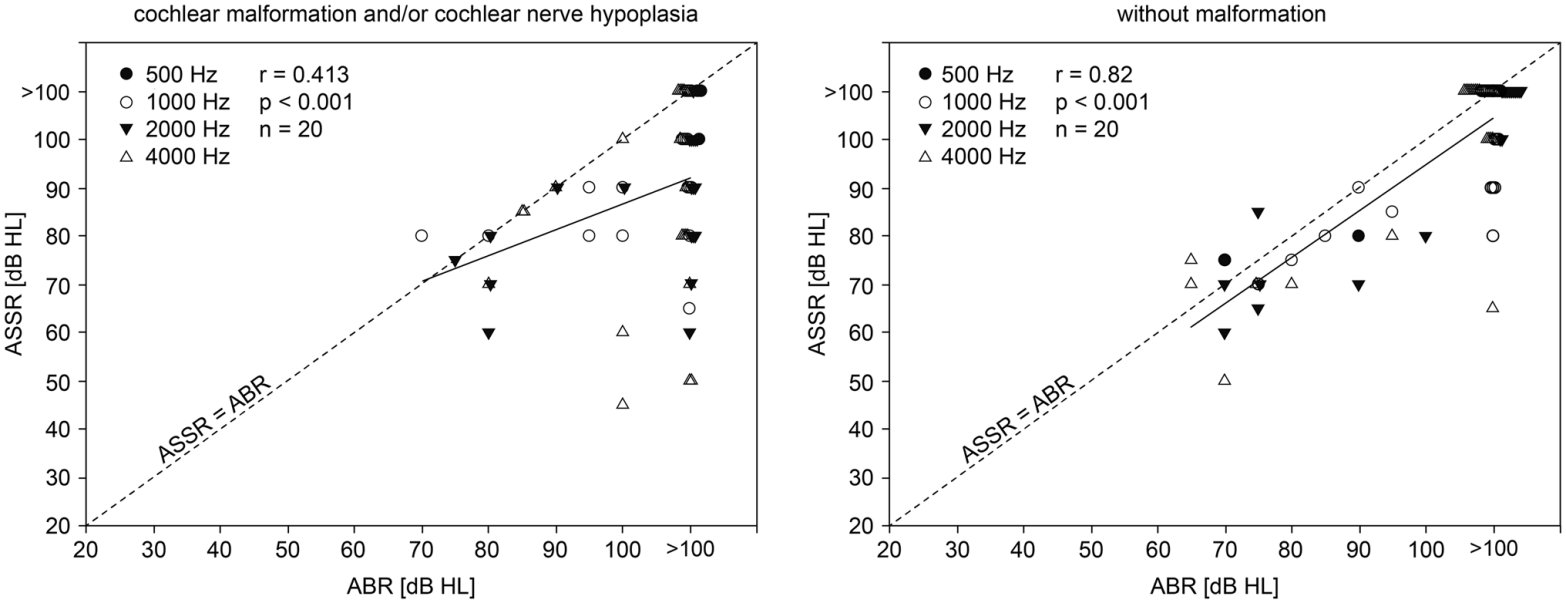
ABR (*x*-axis) and ASSR (*y*-axis) hearing threshold estimations in dB HL correlate in ears with cochlear malformation and/or cochlear nerve hypoplasia taking all value pairs of different center frequencies together, *r* = 0.413 and *p* < 0.001 with *n* = 64 (500 Hz *n* = 4, 1000 Hz *n* = 20, 2000 Hz *n* = 20, 4000 Hz *n* = 20) (left) and ears without malformations, *r* = 0.82 and *p* < 0.001 with *n* = 62 (500 Hz *n* = 2, 1000 Hz *n* = 20, 2000 Hz *n* = 20, 4000 Hz *n* = 20) (right). For visualization of multiple equal value pairs, these are positioned slightly shifted along the *x*-axis. The regression line is drawn as continuous line, for orientation a dotted line is drawn as ASSR = ABR hearing threshold estimation

**Fig. 2:**
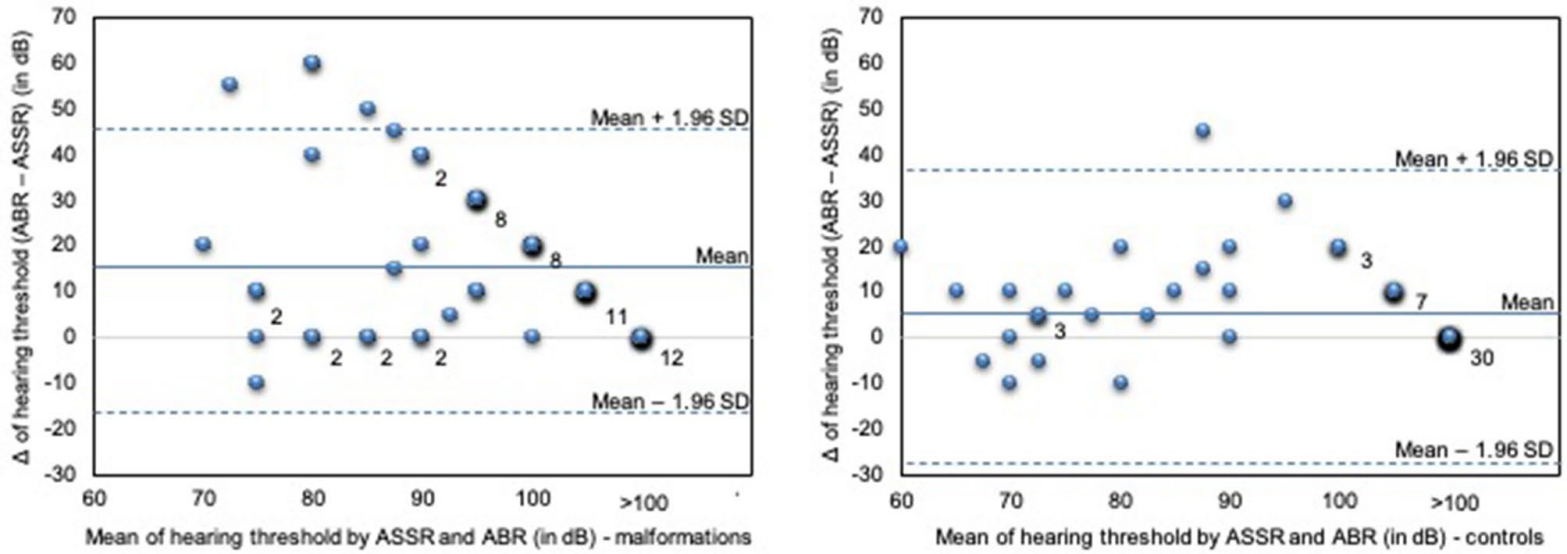
The Bland–Altman-Plot of both evaluated group (controls right and group with cochlear malformation and/or cochlear nerve hypoplasia left) plots the Δ of ABR and ASSR hearing threshold estimations (ABR–ASSR in dB) (*y*-axis) against the corresponding mean value of the measurement methods (*x*-axis). If one data pair is found more than once, the number of equal data pairs is given right next to the data point. The mean ABR–ASSR in dB is drawn as a solid line (in group without malformations mean = 5.3 dB, in the malformation group mean = 15.5 dB), the dashed line shows the range within 1.96 standard deviations (in control group = 16.8, in malformation group = 16.2)

In both patient collectives, ABR and ASSR measurements correlated significantly summarizing value pairs of all CFs as shown in Fig. 1. Nevertheless, in the malformation collective, the correlation coefficient was much lower than in the control group (*r* = 0.413, *p* < 0.001 versus 0.82, *p* < 0.001). In a Bland-Altmann Plot, it becomes obvious, that measurements by ASSR in general estimate better hearing thresholds than by ABR, on the other hand, the difference between ABR and ASSR hearing threshold estimations is lower in ears without malformation (Fig. 2, right) with a mean difference of 5.3 dB compared to ears with CM and/or CNH (Fig. 2, left) with a mean difference of 15.5 dB, although the range of standard deviation is similar (SD in controls = 16.8 dB and in malformations group = 16.1 dB) Also, the distribution of data points—variety of hearing threshold estimations—vary much more in the malformation group than in the control group (Fig. 2).

Table 1 shows a summary of threshold estimations. Depending on the CF, in patients with temporal bone malformations around 31% of hearing threshold estimations by ASSR showed the same result by ABR, whereas in controls without pathological findings in MRI/CT, 52% of measurements led to an equal hearing threshold estimation. This difference was statistically significant (*p* = 0.032). Out of 20 “equal” estimations in 12 (60%) of those cases no hearing threshold could be obtained by neither ABR nor ASSR. Most hearing threshold estimations by ASSR in patients with temporal bone malformations showed a lower/better hearing threshold than estimations by ABR (67% compared to 42% in controls). In 33 of these 43 (78%) cases, no hearing threshold could be obtained by ABR but by ASSR. Only 2% showed a worse hearing threshold estimation in ASSR. 41% of measurements revealed a difference of ≥ 15 dB between ABR and ASSR measurement technique (outliers) in patients with temporal bone malformations compared to 13% in controls (*p* < 0.001).

**Table 1:** Frequency-dependent comparison between malformation and control group

	All CFs collapsed	CF 500 Hz	CF 1000 Hz	CF 2000 Hz	CF 4000 Hz
Temporal bone malformations					
Total no. ears (%)	20 (100%)	4	20	20	20
Data pairs (percent) ASSR threshold ↑	1 (2%)	0 (0%)	1 (5%)	0 (0%)	0 (0%)
** ASSR = ABR**	**20 (31%)**	**3 (75%)**	**5 (25%)**	**4 (20%)**	**8 (40%)**
ASSR threshold ↓	43 (67%)	1 (25%)	14 (70%)	16 (80%)	12 (60%)
** Outliers**	**26 (41%)**	**0 (0%)**	**7 (35%)**	**9 (45%)**	**10 (50%)**
**Controls**					
Total no. ears (%)	20 (100%)	2	20	20	20
Data pairs (percent) ASSR threshold ↑	4 (6%)	1 (50%)	0 (0%)	1 (5%)	2 (10%)
** ASSR = ABR**	32 (52%)*	**0 (0%)**	**11 (55%)**	12 (60%)*	**9 (45%)**
ASSR threshold ↓	26 (42%)	1 (50%)	9 (45%)	7 (35%)	9 (45%)
** Outliers**	8 (13%)*	**0 (0%)**	**4 (20%)**	2 (10%)*	**2 (10%)**

Summarizing all frequencies, only 31% of ears with temporal bone malformation show equal hearing threshold estimation in ASSR and ABR versus in 52% of ears in controls. Correspondingly a much higher percentage of outliers (difference ≥ 15 dB between ABR and ASSR measurement techniques) are found in the malformation group

The bold values are not statistically significant and values marked with a * are significantly different with a *p*-value <.05

*CF* center frequency

*Difference between temporal bone malformations and controls statistically significant

Table 2 shows a list of all individual hearing threshold estimations by ABR and ASSR per ear and grouped for CM, CNH, and combined CM and CNH. Calculating correlation coefficients for ABR hearing threshold estimations and ASSR hearing threshold estimations for the sum of all CFs within the individual subgroups, we could see a remarkable difference for ears with involved CNH: CM (*r* = 0.915; *p* < 0.001), CNH (*r *= 0.534; *p* = 0.003), and CM + CNH (*r* = 0.375; *p* = 0.0919).

**Table 2:** List of individual ABR and ASSR hearing threshold estimations for ears with CM (cochlear malformation), CNH (cochlear nerve hypoplasia) and CM + CNH (combined cochlear malformation and cochlear nerve hypoplasia)

Group	ABR (in dB HL)				ASSR (in dB HL)			
	500 Hz	1000 Hz	2000 Hz	4000 Hz	500 Hz	1000 Hz	2000 Hz	4000 Hz
CM								
1		80	80	85	80	80	80	85
2		–	75	85	100	100	75	85
3		70	80	80	70	80	70	70
4		–	–	–	–	–	–	–
5		–	–	–	–	100	100	100
6	–	–	–	–	–	–	90	–
7		95	90	90	70	90	90	90
8		100	100	100	80	90	90	100
CNH								
1		–	–	–	80	90	80	70
2	–	–	–	–	100	100	100	90
3		–	80	100	85	80	60	45
4		–	–	–	75	65	70	80
5		–	–	–	90	90	90	50
CM + CNH								
1		95	–	100	90	80	80	60
2		100	–	–	80	80	80	80
3		–	–	–	80	90	60	50
4	–	–	–	–	–	–	100	–
5	–	–	–	–	–	–	100	–
6		–	–	–	–	100	100	80
7		–	–	–	100	90	90	80

However, interpreting correlation coefficients on a basis of few data pairs only is limited. Therefore, we looked at two additional parameters to evaluate the individual difference between the groups, which are shown in Table 3. Table 3 shows the patient collective with malformations listed as individual ears in subgroups depending on the type of malformation: CM, CNH and combined malformation CM and CNH. In terms of the type of cochlear malformation, the subgroups CM and CM + CNH did not differ with the same count of IP II, common cavity and cochlear hypoplasia. Two parameters were calculated to show the difference between ABR and ASSR hearing threshold estimation for each ear: the mean of Δ ABR–ASSR (in dB) for CFs 500, 1000, 2000, and 4000 Hz and the number of CFs, where Δ between ABR and ASSR hearing threshold estimation was ≥ 15 dB (outlier).

**Table 3:** Patient data of all four evaluated groups (cochlea malformation (CM), cochlear nerve hypoplasia (CNH), combined cochlear malformation with cochlear nerve hypoplasia (CM + CNH) and controls is listed with age, type of cochlear and nerve malformation (hypoplasia, common cavity, incomplete partition I (IP I), incomplete partition II (IP II)

Individual group with list of patients	Age (in month)	Cochlea malformation	Cochlear nerve	Hearing threshold in ABR/ASSR	Mean of Δ ABR–ASSR (in dB)	No. of outliers
CM						
1	84	Hypoplasia	Normal	+ / +	0	0
2	84	Hypoplasia	Normal	±	3.3	0
3	9	IP I	Normal	+ / +	3.3	0
4	9	com. cavity	Normal	−/−	0	0
5	9	IP II	Normal	−/−	10	0
6	49	IP II	Normal	−/−	6.7	1
7	33	IP II	Normal	−/−	1.7	0
8	33	IP II	Normal	−/−	6.7	0
CNH						
1	12	Normal	Hypoplasia	−/ +	30	3
2	65	Normal	Hypoplasia	−/−	13.3	1
3	69	Normal	Hypoplasia	−/ +	35	3
4	7	Normal	Hypoplasia	−/ +	38.3	3
5	15	Normal	Hypoplasia	−/−	33.3	3
CM + CNH						
1	48	IP II	Hypoplasia	−/ +	28.3	3
2	17	com. cavity	Hypoplasia	−/ +	26.7	3
3	70	IP II	Hypoplasia	−/ +	43.3	3
4	27	Hypoplasia	Hypoplasia	−/−	3.3	0
5	27	Hypoplasia	Hypoplasia	−/−	3.3	0
6	9	IP II	Hypoplasia	−/−	16.6	1
7	9	IP II	Hypoplasia	−/−	23.3	3
Controls						
Sum of 20	31	Normal	Normal	5 × + / +	5.3	1
				2 × −/ +		
				13 × −/−		

Hearing threshold in ABR and ASSR is summarized with “ + ” (in at least 2 center frequencies ≤ 80 dB) and with “−” (in less than 2 center frequencies ≤ 80 dB). Mean values of Δ of hearing threshold by ABR–ASSR are displayed in dB and the number of outliers (Δ ABR–ASSR ≥ 15 dB) is enumerated

Table 3 already shows on an individual basis that the mean value of Δ between ABR and ASSR in dB is much higher in ears with CNH compared to controls or ears with isolated CM. Also, the number of CFs with a Δ ≥ 15 dB was much higher in these patients.

Statistical evaluation by Kruskal–Wallis analysis and group by group comparison with Mann–Whitney rank-sum test revealed statistically significant differences (*p* < 0.05) between isolated CM, CNH and CM + CNH, whereas there was no statistical difference between CM and controls (Table 4). The median of mean Δ of ABR–ASSR hearing threshold estimation in dB for isolated CM and controls were 3.3 dB, for CNH 33.3 and for CM + CNH 23.3 dB. A statistical difference was seen between CM and CNH (*p* = 0.002), CM and CM + CNH (*p* = 0.021), between CNH and controls (*p* = 0.001), and between CM + CNH and controls (*p* = 0.006). We found the same conditions for the number of outliers (Table 4).

**Table 4:** Statistical comparison between the four evaluated groups for median of mean Δ ABR–ASSR (in dB) and for median of number of outliers (Δ ABR–ASSR ≥ 15 dB) for ears with cochlea malformation (CM), cochlear nerve hypoplasia (CNH), combined cochlear malformation with cochlear nerve hypoplasia (CM + CNH) and controls (contr.)

Group	No. of ears	Δ ABR–ASSR (in dB)				No. of outliers			
		Median	*p*-value			Median	*p*-value		
			× CNH	× CM + CNH	× contr		× CNH	× CM + CNH	× contr
CM	8	3.3	**0.002**	0.021	0.979	0	**0.002**	0.029	0.245
CNH	5	33.3		0.202	**0.001**	3		0.432	**0.001**
CM + CNH	7	23.3			**0.006**	3			**0.024**
Contr	20	3.3				0			

*p* values in bold are considered statistically significant

In pairwise multiple comparison procedure (Dunn’s Method), the statistically significant difference (*p* < 0.05) was only seen between CNH and controls and CNH and CM, respectively.

**Fig. 3:**
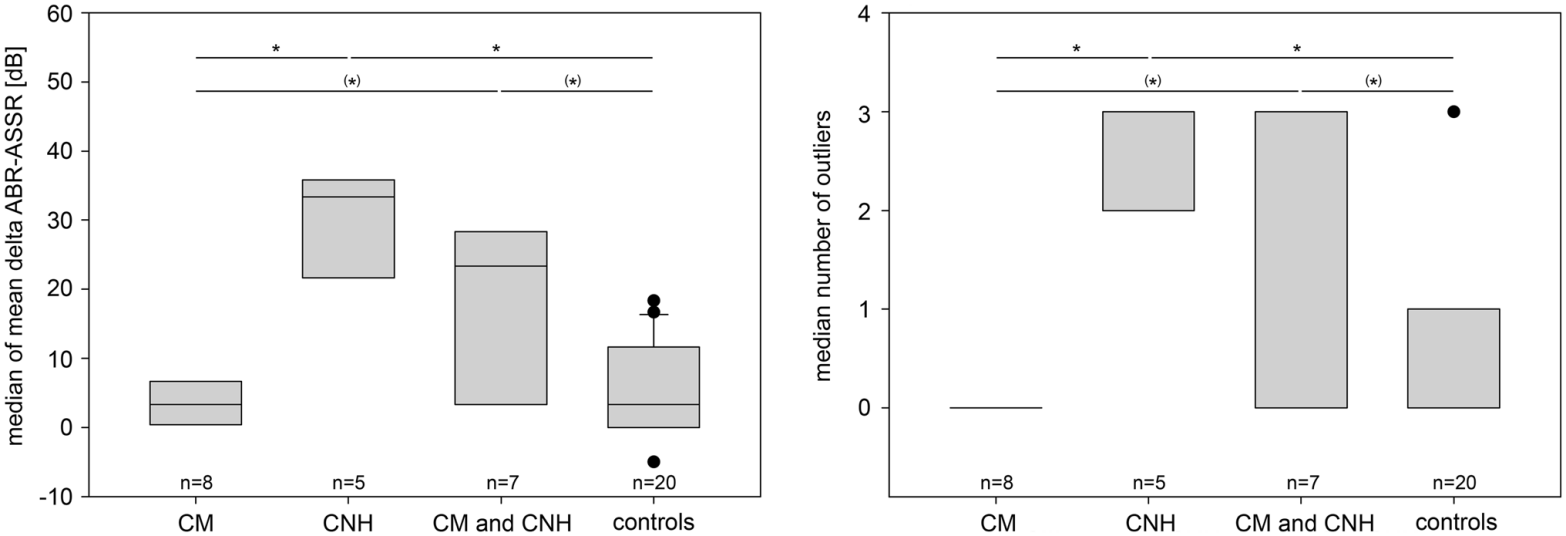
Box plots for each evaluated group (cochlea malformation (CM), cochlear nerve hypoplasia (CNH), combined cochlear malformation with cochlear nerve hypoplasia (CM and CNH) and controls). The difference in the median of delta between ABR–ASSR hearing threshold estimation (in dB) (**a**) and the median in number of outliers (**b**) was plotted with 25% and 75% percentile as lower and upper edge of each column. Statistically significant differences with *p* < 0.05 are labeled with asterisk in case significance was shown in independent rank-sum test and pairwise multiple comparison procedure (Dunn’s method) and labeled with (asterisk) in case significance was only shown in independent rank-sum test

Figure 3 visualizes these differences: median of the delta between ABR and ASSR hearing threshold estimation (a) and median of number of outliers (b) for all four evaluated groups are displayed.

In summary, we could find a markable difference between ABR and ASSR hearing threshold estimation in patients with CNH compared to controls or patients with isolated CM. Hearing threshold estimations were lower (better) by ASSR compared to ABR in these patients.

## Discussion

In this study, we evaluated the influence of CM and CNH on hearing threshold estimation by ABR and ASSR in infants and children with severe or profound hearing loss.

The control group of 20 ears without malformations showed a good correlation of *r *= 0.82 (*p* < 0.001) of ABR and ASSR hearing threshold estimations on all measurements with CFs from 500 Hz to 4 kHz, which is in line with the literature, although these studies used differing stimuli and setups: Rodrigues et al. showed in 17 children with hearing loss correlation coefficients of 0.91, 0.76, 0.81 and 0.89 for 500, 1000, 2000, and 4000 Hz, respectively between ASSR to multiple simultaneous tone-pip stimuli and tone-evoked ABR [6]. A large study on 130 children/260 ears resulted in a correlation of *r* = 0.826 comparing the average of hearing threshold estimation at 1000, 2000, and 4000 Hz by chirp-evoked ASSR and click ABR thresholds [4]. Previous results using the same setup and stimuli could also show this relationship, but on the other hand, indicated individual cases with substantial differences in hearing threshold estimation [19].

In the malformation group in this study, a much lower correlation coefficient of *r* = 0.413 (*p* < 0.001) was revealed between the two techniques (Fig. 1). In general, ASSR hearing threshold estimations were lower (better) than ABR hearing threshold estimations (Table 2; Fig. 2). Hence, the number of equal threshold estimations on a single pair basis was significantly lower in the malformation group compared to the control group (31% versus 52%), as well as the number of outliers—value pairs with a difference of ≥ 15 dB—were significantly higher in the malformation group (41% versus 13%) (Table 1). This could also be visualized in the Bland-Altmann plots (Fig. 2). Comparing the controls (right) with the malformation group (left), the number of ears with higher delta between both techniques is higher in ears with a cochlear and/or nerve malformation. Going into detail, dividing the malformation group into patients/ears with an isolated CM, compared to CNH or combined CM/CNH, we could demonstrate that an isolated CM does not result in a variation in hearing threshold estimation by ABR and ASSR. The median of mean delta between ABR–ASSR estimations and number of outliers was 3.3 dB and 0, respectively, and therefore, comparable to the control group with a median of 3.3 dB and 0 number of outliers (Tables 3, 4; Fig. 3). On the other hand, patients/ears with CNH irrespective of additional CM showed a clinically relevant difference in hearing threshold estimation by ASSR compared to ABR. The median of mean difference was 33.3 dB for isolated CNH and 23.3 dB for combined CM and CNH. Also, this effect was seen over all CFs, the number of outliers was 3 and significantly higher in these patients (Table 4; Fig. 3). The statistical details are summarized in Table 4. Concluding, a relevant difference in hearing threshold estimation by ABR and ASSR with lower (better) estimation by ASSR could indicate a pathology of the cochlear nerve.

Regarding limitations, we would like to address, that this study only included a limited number of patients. We compared patients with malformations with a control group and in both groups’ age and degree of hearing loss were similar but due to the limited number of complete data sets with simultaneous ABR and ASSR measurements in this patient collective both groups could not be directly matched. Also, because of technically limited sound pressure levels, hearing threshold estimations were only obtained up to 100 dB, and beyond, the hearing threshold was set to 110 dB. This imputing technique has been used by other researchers [4], but of course, it causes a blurred delta between measurements in case no hearing threshold can be obtained. On the other hand, it was the only way to deal with this patient population, since malformations including the cochlear nerve mostly fall into the severe/profound hearing loss range. Excluding data pairs without measurable hearing threshold in ABR would have led to only few data pairs in the malformation group, so that statistical analysis would have been impossible. Furthermore, comparing the control group, CM-, CNH- and CM + CNH-group on an individual data pair level excluding data pairs with no measurable hearing threshold in ABR (or vice versa), the same difference would become obvious: the control and CM group had only few outliers versus most data pairs being outliers in the CNH- and CM + CNH-group. Due to a retrospective design, we also had to deal with a limited number of data sets for 500 Hz. Only six data sets (four in the malformation group and two in the control group) were complete in terms of hearing thresholds for both ABR and ASSR measurements. Nevertheless, we did not want to exclude these from the study and as seen in Table 1, these specific six data pairs did not differ considerably, so including these few data sets would not substantially affect statistical analysis. In data comparisons, ASSR threshold estimations at 500 Hz without corresponding ABR data were not included. Also, the retrospective design resulted in the inability to choose procedure parameters in ABR and ASSR measurements. Last, this study concentrated on the comparison of objective hearing threshold estimation by ABR and ASSR rather than comparing objective measurements with behavioral data. Although ABR and ASSR measurements were acquired years ago and patients could have robust behavioral data by now, hearing impairment could have progressed over time and behavioral data were not available in structured age- and time-related context for the study group. Last, hearing impairment involving CNH often presents as single-sided hearing loss, therefore, sufficient masking of the contralateral ear is crucial. In ASSR, masking was done by 70 dB SPL white noise, possibly leading to potential artifacts, on the other hand, this corresponds to the contralateral masking level of 30 dB below stimulation level in ABR at the highest stimulus level of 100 dB.

In the literature, we did not find any study investigating and discussing ASSR hearing threshold estimations in patients with CNH. Only one study mentioned, that ASSR testing was inconclusive in patients with cochlear nerve aplasia without giving further information [20]. Also, Ehrmann-Müller et al. referred to measurable ASSR thresholds in children with CNH and no ABR threshold as a basis for cochlear implantation and investigated the outcome without discussing the discrepancy of ABR and ASSR thresholds [21].

We assume that hearing threshold estimations differ between the techniques, because limited synchrony of the neuronal function could have an individual effect. Whereas chirp-evoked ABR was designed to focus on reproducible peak detection of Jewett wave V depending on time and amplitude [1, 22], ASSR depend on a peak detection across a spectrum [2] and receive contributions from multiple generators [23], maybe masking limited synchrony and thereby estimating a lower (better) hearing threshold than ABR. Also, cochlear nerve hypoplasia in opposition to cochlear nerve aplasia could lead to potential peak detection within statistical limits in ASSR, whereas in ABR, already in cochlear nerve hypoplasia reproducible peak detection cannot be found. We are aware of potential false-positive results in ASSR at levels at high stimulus levels in deaf ears caused by electrical artifacts, on the other hand, in this study, we saw the high discrepancy between ASSR and ABR rather as a systematic finding in a subgroup of the malformation collective than as a random effect, although the numbers of patients were limited in these groups. However, further research is needed to evaluate these effects, the cause of their appearance and investigate, if ASSR actually measure residual hearing that can enable for speech understanding. It might be useful to estimate the hearing threshold in children with proven CNH by MRI using ASSR next to ABR.

In addition, we would like to mention two studies on patients with auditory neuropathy spectrum disorder (ANSD). According to Levi et al., 72% of patients with cochlear nerve dysplasia appear with an audiometric profile of ANSD [24]. Jafari et al. analyzed the correlation between ASSR and behavioral thresholds in 32 ears with ANSD and could not find comparable results. This study did not present ABR thresholds, but the author concluded, that ASSR perhaps could be utilized as an adjunct technique for the differential diagnosis of ANSD [25]. On the contrary, a study evaluating 32 children’s ears with ANSD discussed that ASSR thresholds seemed to reflect the pure tone thresholds despite the preliminary nature of this observation and the need for larger numbers of patients for verification [26]. Therefore, patients without measurable hearing threshold in ABR but detectable otoacoustic emissions should receive MRI in search for a dysplasia of the cochlear nerve. Furthermore, ASSR hearing threshold estimation in these patients with CNH could reveal residual hearing. Especially children with CNH and profound hearing loss in ABR should also receive ASSR measurement for optimal therapeutic strategy and care.

## Conclusion

This study showed a discrepancy in hearing threshold estimation by ASSR and ABR in children with severe or profound hearing loss and hypoplasia of the cochlear nerve itself or in combination with cochlear malformation. At present, it is unclear, how this discrepancy can be explained and whether ASSR or ABR more likely reflect the “real” hearing threshold. More research on comparison with behavioral and psychoacoustic threshold measures is needed to evaluate the reliability of techniques. For now, ASSR and ABR should be jointly used within the diagnostic test battery of children with severe-to-profound hearing loss and CNH.

## Abbreviations

ANSD: Auditory neuropathy spectrum disorder
ABR: Auditory brainstem responses
ASSR: Auditory steady-state response
IP: Incomplete partition
CF: Center frequency
CM: Cochlear malformation
CNH: Cochlear nerve hypoplasia
CT: Computer tomography
EEG: Electroencephalography
MRI: Magnetic resonance imaging

## Declarations

### Availability of data and materials

Data can be received on request.
